# Supplementary material for: An Aedes aegypti-associated fungus increases susceptibility to dengue virus by modulating gut trypsin activity
Source: eLife. 2017 Dec 5;6:e28844. doi: 10.7554/eLife.28844 (PMC5716662; doi:10.7554/eLife.28844)
Supplement: Supplementary file 2. — Sequences underlined corresponds to T7 promoter [file elife-28844-supp2.docx]

**Table 2**: Primer sequences used for dsRNA synthesis and qPCR. Sequences underlined corresponds to T7 promoter

| Name | Primer use |  | Sequence (5’ to 3’) | |
| --- | --- | --- | --- | --- |
| AAEL010196 | T7 | FWD | taatacgactcactatagggCAATGGTTCGCATCATTCTT | |
|  |  | RV | taatacgactcactatagggTTAATCAGTTGACCACCGGAA | |
|  | qPCR | FWD | CGCAAGAGGAATGCCACAAG |  |
|  |  | RV | TTGAATCCAGCGCAGACCAT |  |
| AAEL013714 | T7 | FWD | taatacgactcactatagggCCAAATCTTCAGGTGCGAAT | |
|  |  | RV | taatacgactcactatagggAAGCCTTGTGGCATTCTCTT | |
|  | qPCR | FWD | TTGAAGTGCCCGTTGAGGAA |  |
|  |  | RV | AGCGTTCGGAAAGTAGCGAT |  |
| AAEL013715 | T7 | FWD | taatacgactcactatagggAGATGAACATACGCGTTGGA | |
|  |  | RV | taatacgactcactatagggGGAAGCTCGACAGCGTAGAA | |
|  | qPCR | FWD | TTGGATTGGTGACTCCACGG |  |
|  |  | RV | ATAACCTTCCTCGGCACAGC |  |
| AAEL013707 | T7 | FWD | taatacgactcactatagggACTAAGCATACCCAAGGTGGTC | |
|  |  | RV | taatacgactcactatagggGCAACTCGACAGCGTAGAAA | |
|  | qPCR | FWD | TCGAGGATGGATCGTGCCTA |  |
|  |  | RV | CTTTTCCTGGTTGACTGCGG |  |
| S7 | qPCR | FWD | GCAGACCACCATTGAACACA |  |
|  |  | RV | CACGTCCGGTCAGCTTCTTG |  |
| ITS5 | Sequencing | FWD | TCCTCCGCTTATTGATATGC |  |
| ITS4 |  | RV | GGAAGTAAAAGTCGTAACAAGG | |
